# Evaluation of copper feeding strategies and sources on nursery and finishing pig performance

**DOI:** 10.1093/tas/txag114

**Published:** 2026-07-28

**Authors:** Samantha A Swanson, Jason C Woodworth, Mike D Tokach, Robert D Goodband, Joel M DeRouchey, Katelyn N Gaffield, Jordan T Gebhardt, Roger Cochrane, Matt Einarson

**Affiliations:** Department of Animal Sciences and Industry, College of Agriculture, Kansas State University, Manhattan, KS 66506, United States; Department of Animal Sciences and Industry, College of Agriculture, Kansas State University, Manhattan, KS 66506, United States; Department of Animal Sciences and Industry, College of Agriculture, Kansas State University, Manhattan, KS 66506, United States; Department of Animal Sciences and Industry, College of Agriculture, Kansas State University, Manhattan, KS 66506, United States; Department of Animal Sciences and Industry, College of Agriculture, Kansas State University, Manhattan, KS 66506, United States; Department of Animal Sciences and Industry, College of Agriculture, Kansas State University, Manhattan, KS 66506, United States; Department of Diagnostic Medicine/Pathobiology, College of Veterinary Medicine, Kansas State University, Manhattan, KS 66506, United States; SAM HPRP Chemicals, Inc. DBA SAM Nutrition, Bloomington, MN 55122, United States; SAM HPRP Chemicals, Inc. DBA SAM Nutrition, Bloomington, MN 55122, United States

**Keywords:** copper, copper sulfate, tribasic copper chloride, pigs

## Abstract

Two experiments evaluated the use of approximately 150 mg/kg of added Cu in nursery and finishing pig diets. In Exp. 1, 2204 pigs (initially 5.3 ± 0.09 kg) were used in a 38-d study with 29 pigs/pen and 19 pens/treatment. Pens of pigs were allotted to four feeding strategies in a randomized complete block design. Strategies consisted of feeding duration of 150 mg/kg added Cu: (i) control with 17 mg/kg added Cu, (ii) 150 mg/kg added Cu in phase 3, (iii) 150 mg/kg added Cu in phases 2 and 3, and (iv) 150 mg/kg added Cu in all phases. Treatment diets were fed based on feed budgets of 1.8 kg/pig for phase 1 (d 0 to approximately 10), 5.4 kg/pig for phase 2 (approximately d 10 to 24), and phase 3 was fed for the remainder of the trial (approximately d 24 to 38). Diets within each phase contained either 17 mg/kg (base) or the 17 mg/kg provided by the premix with an additional 150 mg/kg Cu from tribasic copper chloride (NexTrace TBCC, 58%, SAM Nutrition, Bloomington, MN). All diets contained 3000 mg/kg Zn in phase 1 and 2000 mg/kg Zn in phase 2. In phase 2, pigs fed 150 mg/kg added Cu had greater average daily gain (ADG; *P* = 0.012) and average daily feed intake (ADFI; *P* = 0.021) than pigs fed the control diet. However, no differences were observed among strategies for overall performance. In Exp. 2, 2160 pigs (initially 24.1 ± 0.60 kg) were used in a 110-d study with 27 pigs/pen and 20 pens/treatment. Pens of pigs were allotted to one of four treatments: base level of Cu or 150 mg/kg Cu from TBCC source 1 (NexTrace TBCC, 58%, SAM Nutrition, Bloomington, MN), TBCC source 2 (Intellibond C, 54%, Selko, Tilburg, Netherlands), and CuSO_4_ (25.2%, Phibro Animal Health Corp., Teaneck, NJ). All diets contained basal Cu from the premix with those containing added Cu containing an additional 150 mg/kg Cu from treatment sources. From d 0 to 56, pigs fed added Cu had greater ADG (*P* < 0.001) and a tendency for increased ADFI and G:F (*P* ≤ 0.059) with no source differences. From d 56 to 110, no differences in growth were observed. Overall, the addition of 150 mg/kg Cu increased ADG (*P* = 0.018) and final weight (*P* = 0.008) and a tendency for increased ADFI (*P* = 0.072); however, there were no differences observed between Cu source. In summary, 150 mg/kg added Cu provided limited overall benefit in the nursery when 2000 to 3000 mg/kg of Zn were fed. In grow-finish pig diets, 150 mg/kg of added Cu improved growth performance regardless of source, with the greatest response during the early growing phase.

## Teaser

The addition of 150 mg/kg of Cu provided limited overall benefit in nursery pigs. However, in grow-finish pig diets, 150 mg/kg of added Cu improved growth performance, with the greatest response observed in the early growing period.

## Introduction

Copper (Cu) is generally added to swine diets because it is needed for processes involving growth, development, and maintenance (Gaetke and Chow 2003). There are a variety of sources of Cu available for use in swine diets. Common sources used include copper sulfate (CuSO_4_), tribasic copper chloride (TBCC), chelated Cu, and others. The NRC (2012) describes the dietary copper requirement estimate for pigs weighing between 5 and 130 kg as 3 to 6 mg/kg. However, previous research has observed improvements in growth performance when high levels (100 to 250 mg/kg) of Cu are included in the diet (Cromwell et al. 1998; Hill et al. 2000; Hastad et al. 2001).

A review of different levels and sources of added Cu in nursery diets identified that the inclusion of high levels of added Cu, independent of source, increased average daily gain (ADG) and gain:feed ratio (G:F; Galiotto Miranda et al. 2024). Within commercial production, it is common for 150 to 250 mg/kg of Cu to be added in late nursery diets (Faccin et al. 2023). However, limited research has been conducted in recent years evaluating the most beneficial phase feeding strategies for high levels of added Cu when fed in combination with 2000 to 3000 mg/kg of Zinc (Zn). Some research indicates there are no additive effects when high levels of Zn and Cu are used together (Smith et al. 1997). However, Pérez et al. (2011) indicated that high levels of both Zn and Cu were additive in promoting the growth of weaned pigs. Therefore, the objective of Exp. 1 was to compare the effects of feeding 150 mg/kg of added Cu, provided by TBCC, throughout three nursery phases on growth performance when diets included 2000 to 3000 mg/kg Zn from ZnO.

Within growing-finishing pigs, the benefits from 75 to 150 mg/kg of added Cu are typically observed in the early growing period (NCR-42 1974; Hastad et al. 2001), although some research has also observed positive effects in later finishing stages. Coble et al. (2017) demonstrated a linear improvement in growth performance and hot carcass weight (HCW) in finishing pigs when supplementing diets with 75 mg/kg or 150 mg/kg Cu from either CuSO_4_ or TBCC. Primarily, previous research has compared TBCC to CuSO_4_, with little research directly comparing different sources of TBCC. As more Cu sources become available, further research is needed to characterize their response in swine diets. Therefore, the objective of Exp. 2 was to compare the effects of three Cu sources fed at 150 mg/kg (TBCC source 1, TBCC source 2, or CuSO_4_) compared to a control diet containing a base level of Cu provided from the trace mineral premix on growth performance and carcass characteristics in finishing pigs.

## Materials and methods

### Animal facilities

The Kansas State University Institutional Animal Care and Use Committee approved the protocol used in these experiments (IACUC # 4847). Experiment 1 was conducted in two rooms at a commercial nursery research site (Hord Farms West, Pipestone, MN). Each pen (2.3 × 3.7 m) provided approximately 0.32 m^2^ per pig and was equipped with a 6-hole dry feeder and a drinker pan. Experiment 2 was conducted in two independent barns at a commercial finishing research site (Hord Farms West, Pipestone, MN). Finishing barns were naturally ventilated and double-curtain-sided with totally slatted floors. Each pen (3.0 × 5.6 m) provided approximately 0.63 m^2^ per pig and was equipped with a 5-hole dry feeder and a cup drinker. In both facilities, daily feed additions were recorded using a computerized feeding system (FeedPro; Feedlogic Corp., Willmar, MN). Pigs were provided ad libitum access to feed and water throughout both experiments.

### Animals and diets

#### Exp. 1

A total of 2204 weanling pigs (PIC 337 × 1050, initially 5.3 ± 0.09 kg) were used in a 38-d study with 29 pigs per pen and 19 pens per treatment. Pigs were weaned at approximately 20 d of age and randomly allotted to pens. Pens of pigs were blocked by initial body weight (BW) and allotted to one of four feeding strategies in a randomized complete block design.

The four Cu feeding strategies evaluated consisted of an added 150 mg/kg of Cu in the different nursery phases. Treatment diets were formulated in three phases and fed based on feed budgets of 1.8 kg/pig for phase 1 (d 0 to approximately 10), 5.4 kg/pig for phase 2 (approximately d 10 to 24), and phase 3 was fed for the remainder of the trial (approximately d 24 to 38). Feeding strategy 1 served as the control with 17 mg/kg of added Cu provide by the trace mineral premix, strategy 2 included an added 150 mg/kg of Cu in only phase 3, strategy 3 consisted of an added 150 mg/kg of Cu in phases 2 and 3, and strategy 4 included an added 150 mg/kg of Cu in all three phases (Table 1). Within each experimental phase, there were two diets provided, either a base level of Cu (17 mg/kg) or an added 150 mg/kg of Cu on top of that provided from the trace mineral premix (167 total added). The base level of Cu was provided from CuSO_4_ in the vitamin/trace mineral premix. In phases where an additional 150 mg/kg of Cu were fed, TBCC (NexTrace TBCC, 58%, SAM Nutrition, Bloomington, MN) was added to diets as a separate ingredient. Complete feed samples were analyzed for Cu in duplicate (Table 2; AOAC 2000, method 985.01; Cumberland Valley Analytical Services, Inc., Waynesbro, PA).

**Table 1:** Composition of experimental diets, Exp. 1 (as-fed basis)^1^.

Item	Phase 1	Phase 2	Phase 3
**Ingredient, %**			
** Corn**	40.57	57.63	57.42
** Soybean meal, 47.7% CP**	17.76	29.92	28.53
** Whey powder**	25.00	–	–
** Dried distillers grains with solubles**	5.00	7.50	10.00
** Microbial enhanced soybean meal^2^**	5.00	–	–
** Corn oil**	2.50	–	–
** Calcium carbonate**	0.45	0.75	0.85
** Monocalcium phosphate, 21% P**	1.10	1.10	0.70
** Sodium chloride**	0.15	0.45	0.29
** Liquid lysine, 55%^3^**	–	0.75	0.70
** L-Lys-HCl**	0.45	–	–
** DL-Met**	0.26	0.23	0.19
** L-Thr**	0.18	–	–
** Thr^4^**	–	0.28	0.24
** L-Trp**	0.03	0.02	–
** L-Val**	0.11	0.11	0.08
** Vitamin premix^5^**	0.25	0.25	0.25
** Trace mineral premix^6^**	0.15	0.15	0.15
** Choline chloride, 60%**	0.04	–	–
** Phytase^7^**	0.06	0.06	0.06
** Zinc oxide**	0.40	0.26	–
** Mycotoxin binder^8^**	0.55	0.55	0.55
** TBCC, 58%^9^**	+/-	+/-	+/-
**Total**	100	100	100
**Calculated analysis**			
**Standardized ileal digestible (SID) amino acids, %**			
** Lys**	1.35	1.35	1.30
** Ile:Lys**	59	56	58
** Leu:Lys**	120	122	129
** Met:Lys**	39	39	38
** Met and Cys:Lys**	60	60	60
** Thr:Lys**	65	65	65
** Trp:Lys**	20.3	20.3	20.6
** Val:Lys**	70	70	70
** His:Lys**	34	37	39
**Net energy, kcal/kg**	2578	2376	2394
**Crude protein, %**	20.7	22.2	22.2
**Ca, %**	0.72	0.71	0.68
**STTD P, %^10^**	0.62	0.51	0.44
**Ca:P**	1.04	1.10	1.20
**Zn, mg/kg**	3000	2000	110

1 Phase 1 and 2 diets were provided using a feed budget of 1.8 and 5.4 kg/pig, phase 3 was fed for the remainder of the trial.

2 ME-PRO, Prairie Aquatech, Brookings, SD.

3 L-Lysine 55% liquid CJ America Bio, Downers Grove, IL.

4 ThrPro CJ America Bio, Downers Grove, IL.

5 Provided per kg of feed: 4134 IU of vitamin A as retinyl acetate; 1653 IU of vitamin D3 as cholecalciferol; 44 IU vitamin E as dl-α-tocopherol acetate; 3 mg of vitamin K as menadione; 0.03 mg of vitamin B12 as cyanocobalamin; 50 mg of niacin; 26 mg of pantothenic acid as d-calcium pantothenate; 8 mg of riboflavin.

6 Provided per kg of feed: 17 mg of Cu as copper sulfate; 0.3 mg I as ethylenediamine dihydriodide; 110 mg Fe as ferrous sulfate; 33 mg Mn as manganese oxide; 0.3 mg Se as sodium selenite; 110 mg of Zn as zinc sulfate.

7 HiPhorius 2400 (DSM-Firmenich, Maastricht, Netherlands) was added at 1500 FYT/kg and provided an estimated release of 0.12% STTD P.

8 Feed Aid NutriQuest, Mason City, IA.

9 Tribasic copper chloride, (NexTrace TBCC, 58%, SAM Nutrition Bloomington, MN) added at 0.03% of the diet was included at the expense of corn to provide 150 mg/kg of Cu.

10 Standardized total tract digestible phosphorus.

**Table 2:** Analyzed Cu concentration of experimental diets, Exp. 1 (as-fed basis)^1^.

	Cu, mg/kg^2^
**Phase 1**	
** Base level Cu**	29
** Added 150 mg/kg Cu**	172
**Phase 2**	
** Base level Cu**	27
** Added 150 mg/kg Cu**	171
**Phase 3**	
** Base level Cu**	28
** Added 150 mg/kg Cu**	195

1 Values represent the mean of two samples analyzed in duplicate (Cumberland Valley Analytical Services, Inc., Waynesboro, PA).

2 Tribasic copper chloride (NexTrace TBCC, 58%, SAM Nutrition, Bloomington, MN) provided an additional 150 mg/kg of Cu (167 mg/kg total added Cu).

Diets in phases 1 and 2 provided added Zn above basal nutritional levels (approximately 3000 mg/kg Zn in phase 1 and approximately 2000 mg/kg Zn in phase 2). Phase 3 contained 110 mg/kg Zn. Phase 1 diets were manufactured at Hubbard Feeds in Mankato, MN. Phase 2 and 3 diets were manufactured at the Hord Farms West feed mill located in Pipestone, MN. The first phase was fed in pellet form, and the remaining two phases were fed in meal form. Pen weights and feed disappearance were recorded on d 10, 17, 24, 31, and 38 to determine ADG, average daily feed intake (ADFI), and G:F. Feed delivered was recorded for each pen, with any feed remaining in feeders recorded on each weigh day by a feeder measurement. Mortality and removals were recorded for the duration of the trial. Under the circumstance a pig died or was removed from the study due to inability to overcome sickness or injury, the weight of the pig and feed consumption up until that date was recorded.

#### Exp. 2

A total of 2160 pigs (PIC 337 × 1050; initially 24.1 ± 0.60 kg) were used in a 110-day study with 27 pigs per pen and 20 pens per treatment. Pigs were randomly assigned to a pen upon arrival in the barn and fed a common diet containing approximately 145 mg/kg added Cu until they reached approximately 23 kg. Pens of pigs were then blocked by weight and randomly allotted to one of four dietary treatments.

Dietary treatments consisted of a control diet containing a base level of Cu (ranging from 16 to 11 mg/kg Cu), or the base level with an additional 150 mg/kg of Cu from one of three sources: TBCC source 1 (NexTrace TBCC, 58%, SAM Nutrition, Bloomington, MN), TBCC source 2 (Intellibond C, 54%, Selko, Tilburg, Netherlands), and CuSO_4_ (25.2%, Phibro Animal Health Corp., Teaneck, NJ; Table 3). All treatment diets contained a base level of CuSO_4_ provided by the trace mineral premix, which provided 16 mg/kg Cu in phases 1 and 2, 13 mg/kg Cu in phase 3, and 11 mg/kg Cu in phase 4 based on a tapered inclusion of the trace mineral premix throughout the experiment. An additional 150 mg/kg Cu was added from the other three sources for their respective dietary treatment. Complete feed samples were analyzed for Cu in duplicate identical as outlined for Exp. 1 (Table 4).

**Table 3:** Composition of experimental diets, Exp. 2 (as-fed basis)^1^.

Item	Phase 1	Phase 2	Phase 3	Phase 4
**Ingredient, %**				
** Corn**	57.53	62.81	67.87	85.56
** Soybean meal, 47.7% CP**	19.48	14.36	9.59	12.23
** Dried distiller’s grains with solubles**	20.00	20.00	20.00	–
** Calcium carbonate**	1.38	1.35	1.28	0.93
** Salt**	0.35	0.35	0.35	0.35
** Monocalcium phosphate, 21% P**	0.23	0.20	0.05	0.23
** Liquid Lys, 55%^2^**	0.66	0.62	0.59	0.44
** DL-Met**	0.05	0.01	–	0.02
** Thr^3^**	0.14	0.12	0.10	0.11
** L-Trp**	0.04	0.04	0.04	0.03
** Vitamin/trace mineral premix^4^**	0.11	0.11	0.10	0.08
** Phytase^5^**	0.05	0.05	0.05	0.05
** Cu Source^6^**	+/-	+/-	+/-	+/-
**Total**	100	100	100	100
**Calculated analysis**				
**Standardized ileal digestible (SID) amino acids, %**				
** Lys**	1.10	0.95	0.82	0.72
** Ile:Lys**	60	60	60	60
** Leu:Lys**	148	159	171	153
** Met:Lys**	31	30	31	30
** Met and Cys:Lys**	57	57	60	58
** Thr:Lys**	64	64	65	65
** Trp:Lys**	19.1	19.1	19.0	19.3
** Val:Lys**	69	72	74	70
** His:Lys**	42	43	45	43
** Net energy, kcal/kg**	2394	2424	2460	2552
** Crude protein, %**	20.2	18.1	16.2	13.2
** Ca, %**	0.62	0.59	0.52	0.43
** STTD P, %^7^**	0.39	0.37	0.33	0.27
** Ca:P**	1.20	1.21	1.20	1.20

1 Phase one was fed from approximately 23 to 50 kg, phase 2 from 50 to 75 kg, phase 3 from 75 to 100 kg, and phase 4 from 100 kg to marketing at approximately 127 kg.

2 L-Lys 55% liquid CJ America Bio, Downers Grove, IL.

3 ThrPro CJ America Bio, Downers Grove, IL.

4 Provided per kg of premix: 2,645,520 IU of vitamin A as retinyl acetate; 1,047,185 IU of vitamin D3 as cholecalciferol; 27,778 IU vitamin E as dl-α-tocopherol acetate; 2160 mg of vitamin K as menadione; 22 mg of vitamin B12 as cyanocobalamin; 35,494 mg of niacin; 17,637 mg of pantothenic acid as d-calcium pantothenate; 5070 mg of riboflavin; 14,545 mg of Cu as copper sulfate; 318.18 mg I as ethylenediamine dihydriodide; 88,182 mg Fe as ferrous sulfate; 20,909 mg Mn as manganese oxide; 236.36 mg Se as sodium selenite; 88,182 mg of Zn as zinc sulfate.

5 Quantum Blue 2G (AB Vista, Marlborough, England) was added at 1016 FTU/kg and provided an estimated release of 0.12% STTD P.

6 Cu sources were included at 0.026%, 0.028% and 0.06% of the diet for NexTrace TBCC, 58% (SAM Nutrition, Bloomington, MN), Intellibond C, 54% (Selko, Tilburg, Netherlands) and CuSO_4_ (25.2%, Phibro Animal Health Corp., Teaneck, NJ) at the expense of corn to provide an additional 150 mg/kg of Cu.

7 Standardized total tract digestible phosphorus.

**Table 4:** Analyzed Cu concentration of experimental diets, Exp. 2 (as-fed basis)^1^.

	Phase 1				Phase 2				Phase 3				Phase 4			
	Base level Cu	TBCC Source 1	TBCC Source 2	CuSO_4_	Base level Cu	TBCC Source 1	TBCC Source 2	CuSO_4_	Base level Cu	TBCC Source 1	TBCC Source 2	CuSO_4_	Base level Cu	TBCC Source 1	TBCC Source 2	CuSO_4_
**Cu, mg/kg**	62	188	183	159	27	196	172	190	47	196	180	185	46	145	198	184

1 Values represent the mean of two samples analyzed in duplicate (Cumberland Valley Analytical Services, Inc., Waynesboro, PA). All treatments contained a base level of CuSO_4_ provided by the trace mineral premix. Inclusions were 16 mg/kg Cu in phases 1 and 2, 13 mg/kg Cu in phase 3, and 11 mg/kg Cu in phase 4 based on a tapered inclusion of the vitamin/trace mineral premix throughout the experiment. In addition to the base level of Cu in all diets, treatments consisted of adding 150 mg/kg of the respective Cu source, TBCC source 1 (NexTrace TBCC, 58%, SAM Nutrition, Bloomington MN), TBCC source 2 (Intellibond C, 54%, Selko, Tilburg, Netherlands), or CuSO_4_ (25.2%, Phibro Animal Health Corp., Teaneck, NJ) on top of the base Cu level. Treatment diets were formulated in four phases: phase one from approximately 23 to 50 kg, phase 2 from 50 to 75 kg, phase 3 from 75 to 100 kg, and phase 4 from 100 kg to marketing at approximately 127 kg.

Test ingredients were mixed into hand-add packs using approximately 0.9 kg of ground corn per ton of feed. All diets were fed in meal form and manufactured at the Hord Farms West feed mill located in Pipestone, MN. Treatment diets were formulated in four phases and fed based on average barn weight: phase one, approximately 23 to 50 kg, phase two from 50 to 75 kg, phase three from 75 to 100 kg, and phase four from 100 kg to marketing at approximately 127 kg. Phases 1 and 2 were combined to form the grower phase (d 0 to 56) and phases 3 and 4 were combined to represent the finisher phase (d 56 to 110). Pen weights and feed disappearance were recorded approximately every 14 d to determine ADG, ADFI, and G:F. Feed deliveries were recorded by pen, and residual feed was measured on each weigh day. Mortality and removals were recorded throughout the trial. For pigs that died or were removed due to illness or injury, body weight and feed intake were recorded up to the time of removal.

### Carcass data

In Exp. 2, when the barn average pig weight reached approximately 110 kg, the three heaviest pigs per pen were selected and marketed at a United States Department of Agriculture (USDA) inspected processing facility (JBS Foods, Worthington, MN). No carcass data were collected during this first marketing event. At the end of the study, approximately two to three weeks after the first marketing event, depending on barn, the remaining pigs were marketed. Standard carcass measurements of backfat depth, loin depth, percentage lean, and hot carcass weight (HCW) were collected for each individual carcass and summarized by pen. The percentage lean calculations were obtained from a proprietary equation used by the packer. Carcass yield was calculated by dividing the pen average HCW by the pen average final live weight obtained at the farm.

### Statistical analysis

Data were analyzed in R studio (Version 4.3.1 R Core Team, Vienna, Austria) as a one-way ANOVA using the lmer function, including treatment as a fixed effect and weight block as a random effect with the addition of barn serving as a random effect in Exp. 2. Hot carcass weight was used as a covariate for carcass characteristics. In Exp. 1, contrasts were used to test for the main effects of Cu level within each phase. In Exp. 2, contrasts were used to test for the effect of base level Cu vs an added 150 mg/kg of Cu and between Cu sources. Means separation was performed using a Tukey multiple comparison adjustment using the emmeans package. *P*-values were considered significant at *P* < 0.05 and tendencies at 0.05 < *P* ≤ 0.10.

## Results

Analysis of complete diets for both experiments indicated that analyzed Cu levels were within the expected range of formulated levels (Tables 2 and 4). No meaningful deviations from the expected Cu levels were observed when accounting for analytical variation and Cu levels coming from basal ingredients, nor were any observed for other nutrients.

### Exp. 1

In phase 1 (approximately d 0 to 10), there was no evidence of a difference in performance observed between pigs fed the base level of Cu and pigs fed a diet containing an additional 150 mg/kg of Cu (Table 5). In phase 2 (approximately d 10 to 24), pigs fed an additional 150 mg/kg of Cu had greater ADG (*P* = 0.012) and ADFI (*P* = 0.021) compared to pigs fed the base level of Cu, with no evidence of a difference for G:F. Additionally, pigs fed an additional 150 mg/kg of Cu in phases 1 and 2 had greater ADG during phase 2 than those fed a base level of Cu in phase 1 and 2 (*P* = 0.047), with pigs fed an additional 150 mg/kg of Cu in only phase 3 and pigs fed added Cu in phases 2 and 3 intermediate.

**Table 5:** Effects of copper feeding strategy on growth performance of nursery pigs, Exp. 1^1^.

	Cu, mg/kg						
Phase 1:	17	17	17	150		*P =*	
Phase 2:	17	17	150	150		Treatment	Base vs 150 mg/kg added Cu^2^
Phase 3:	17	150	150	150	SEM		
**BW, kg**							
** d 0**	5.3	5.3	5.3	5.3	0.09	0.998	–
** d 10^3^**	6.6	6.4	6.4	6.4	0.10	0.034	0.250
** d 24**	11.8	11.6	11.7	11.9	0.19	0.503	0.327
** d 38**	18.3	18.2	18.5	18.5	0.22	0.341	0.636
**Phase 1**							
** ADG, g**	103	95	94	89	5.1	0.206	0.129
** ADFI, g**	171	165	167	164	4.4	0.193	0.162
** G:F, g/kg**	602	568	564	547	26.3	0.501	0.302
**Phase 2**							
** ADG, g**	358^b^	364^ab^	370^ab^	376^a^	7.8	0.047	0.012
** ADFI, g**	476	478	488	495	8.7	0.108	0.021
** G:F, g/kg**	753	761	759	760	8.4	0.857	0.748
**Phase 3**							
** ADG, g**	453	451	465	454	13.8	0.515	0.644
** ADFI, g**	735	719	741	745	17.6	0.336	0.976
** G:F, g/kg**	614	627	628	609	9.7	0.141	0.360
**Overall**							
** ADG, g**	314	310	317	314	5.8	0.613	–
** ADFI, g**	474	464	475	478	7.8	0.273	–
** G:F, g/kg**	662	668	667	657	5.1	0.237	–
**Removals, %**	20.8	21.0	22.3	21.5	2.29	0.936	–
**Mortality in test pen, %**	1.6	2.2	2.2	3.1	0.74	0.450	–

a,b Means in the same row with different superscripts differ (*P* < 0.05).

1 A total of 2204 (PIC 1050 × 337; initially 5.3 ± 0.09 kg) were used in a 38-d trial with 29 pigs per pen and 19 replications per treatment. All treatment diets contained 17 mg/kg of Cu from the trace mineral premix. Treatments consisted of added 150 mg/kg on top of the base Cu level for differing feeding strategies by phase. Treatment diets were fed based on a feed budget of 1.8 kg/pig for phase 1 (approximately d 0 to 10), 5.4 kg/pig for phase 2 (approximately d 10 to 24), and phase 3 (approximately d 24 to 38) was fed for the remainder of the trial.

2 Compares the means of treatments with a base level of Cu provided in the trace mineral premix (17 mg/kg) to treatments containing an additional 150 mg/kg of added Cu (167 total) within phase.

3 Although a significant treatment P-value, means did not separate using a Tukey multiple means separation test.

In phase 3 (approximately d 24 to 38), there were no differences observed in growth performance between pigs fed the base level of Cu and an additional 150 mg/kg of Cu, nor any differences in any of the feeding strategies. There were no differences between Cu feeding strategies in the overall growth performance (d 0 to 38) or removals and mortality.

### Exp. 2

There was no evidence of difference observed among Cu sources when fed at an additional 150 mg/kg for any response criteria. In the grower period (d 0 to 56), pigs fed an additional 150 mg/kg of Cu had a greater (*P* < 0.001) BW and ADG compared to pigs fed the control diet containing the basal level of Cu (Table 6). A tendency (*P* ≤ 0.059) was observed in pigs fed an additional 150 mg/kg of Cu for increased ADFI and G:F when compared to pigs fed the control diet. In the finishing period (d 56 to the end of the trial), there was no evidence of differences observed in ADG, ADFI or G:F when comparing pigs fed an additional 150 mg/kg of Cu to those fed the control diet.

**Table 6:** Effects of copper source on finishing pig growth performance and carcass characteristics, Exp. 2^1^.

		Added 150 mg/kg Cu				*P =*	
	Base level Cu	TBCC Source 1	TBCC Source 2	CuSO_4_	SEM	Treatment	Base level vs 150 mg/kg Cu^2^
**BW, kg**							
** d 0**	24.1	24.1	24.1	24.1	0.60	0.998	–
** d 56^3^**	76.7	77.9	78.2	78.2	1.31	0.001	< 0.001
** Final**	125.8	127.9	128.0	127.6	1.36	0.063	0.008
**Grower^4^**							
** ADG, g^3^**	933	953	960	956	14.8	0.004	< 0.001
** ADFI, g**	2105	2121	2150	2143	48.6	0.131	0.059
** G:F, g/kg**	445	451	448	448	4.0	0.186	0.057
**Finisher^5^**							
** ADG, g**	949	957	961	949	27.1	0.686	0.462
** ADFI, g**	2960	2981	3010	2987	53.7	0.468	0.203
** G:F, g/kg**	319	320	319	317	4.1	0.671	0.683
**Overall^6^**							
** ADG, g**	938	953	959	951	10.4	0.087	0.018
** ADFI, g**	2507	2526	2554	2539	26.3	0.185	0.072
** G:F, g/kg**	374	378	376	375	3.0	0.410	0.338
**Carcass characteristics**							
** HCW, kg^7^**	93.8	95.0	95.2	94.8	0.98	0.171	0.031
** Yield, %**	74.1	73.8	74.0	74.0	0.22	0.671	0.363
** Backfat, mm**	16.72	16.56	16.77	16.47	0.320	0.590	0.543
** Loin depth, mm**	68.15	67.87	67.80	67.90	0.620	0.888	0.447
** Lean, %**	56.86	56.94	56.78	56.99	0.224	0.622	0.733
**Removals and mortality, %**							
** Removals**	4.26	3.33	3.52	4.44	0.887	0.733	0.586
** Mortality**	0.56	1.30	1.30	1.85	0.580	0.330	0.113
** Removals and mortality**	4.81	4.63	4.81	6.30	1.045	0.574	0.725

1 A total of 2160 pigs (PIC 1050 × 337; initially 24.1 ± 0.60 kg) were used in a 110-d trial with 27 pigs per pen and 20 replications per treatment using two research barns. All treatments contained a base level of CuSO_4_ provided by the trace mineral premix. Inclusions were 16 mg/kg Cu in phases 1 and 2, 13 mg/kg Cu in phase 3, and 11 mg/kg Cu in phase 4 based on a tapered inclusion of the vitamin/trace mineral premix throughout the experiment. In addition to the base level of Cu in all diets, treatments consisted of adding 150 mg/kg of the respective Cu source, TBCC source 1 (NexTrace TBCC, 58%, SAM Nutrition, Bloomington MN), TBCC source 2 (Intellibond C 54%, Selko, Tilburg, Netherlands), or CuSO_4_ (25.2%, Phibro Animal Health Corp., Teaneck, NJ) on top of the base Cu level. Treatment diets were formulated in four phases: phase one from approximately 23 to 50 kg, phase 2 from 50 to 75 kg, phase 3 from 75 to 100 kg, and phase 4 from 100 kg to marketing at approximately 127 kg.

2 Compares the control treatment containing a base level of Cu with the mean of treatments fed 150 mg/kg of Cu within the period. Contrasts comparing Cu sources were not significant (*P* > 0.10) throughout the entire trial for any response criteria.

3 Although a significant treatment P-value, means did not separate using a Tukey multiple means separation test.

4 Grower period represents d 0 to 56.

5 Finisher period represents d 56 to 110.

6 Overall period represents d 0 to 110.

7 Hot carcass weight (HCW) served as a covariate for carcass data.

Overall, pigs fed an additional 150 mg/kg of Cu had greater (*P* = 0.018) ADG and final BW (*P* = 0.008) when compared to pigs fed the base level of Cu. There was a tendency observed for greater (*P* = 0.072) ADFI for pigs fed an additional 150 mg/kg of Cu compared to pigs fed the base level of Cu. Hot carcass weight was greater (*P* = 0.031) in pigs fed an additional 150 mg/kg of Cu than pigs fed the base level of Cu. No evidence of any differences was observed for other carcass characteristics or removals and mortality.

## Discussion

The NRC (2012) estimated Cu requirement for pigs is 3 to 6 mg/kg. The control diets with only 13 to 17 mg/kg of added Cu in the trace mineral premixes for both of our experiments contained levels above this to ensure the basic requirement was met. Additional levels of Cu of 100 to 250 mg/kg have been known to improve growth performance (Hastad et al. 2001; Huang et al. 2015; Espinosa et al. 2017). Some researchers have attributed the improvement in performance to antimicrobial effects (Shurson et al. 1990). Jensen (2016) concluded that Cu supplementation, even in small amounts (less than 50 mg/kg), seemed to reduce the population of clostridia and coliform bacteria, whereas high levels (greater than 170 mg/kg) seem to reduce the presence of lactobacillus. However, others have suggested Cu impacts growth and metabolism in other ways. High (75 to 250 mg/kg) levels of Cu often increase ADG and ADFI. Zhou et al. (1994) observed greater concentrations of growth hormones in the pituitary gland of pigs fed 215 mg/kg added Cu. Dove (1995) suggested that these levels of Cu may improve fat digestibility. Li et al. (2008) observed that high dietary Cu increased neuropeptide Y concentrations and neuropeptide Y mRNA expression in the hypothalamus, which may be responsible for the increased feed intake and gain observed with high Cu addition.

Within swine diets, commonly used Cu sources include CuSO_4_, TBCC, chelated Cu and others. Copper sulfate is manufactured using Cu metal and sulfuric acid and is highly soluble in water (Miles et al. 1998). Tribasic copper chloride is a by-product of the manufacturing process of circuit boards, where acidic cupric chloride and alkaline cupramine chloride solution are neutralized to form a green purified source of Cu (Coble et al. 2017). This source is less than 1% soluble in water (Miles et al. 1998). Due to the differences in manufacturing, the concentration of Cu in TBCC is greater than that of CuSO_4_, with concentrations of 54 to 58% Cu in TBCC compared to 25.2% Cu in CuSO_4_. Tribasic copper chloride has also been shown to have less oxidative instability during storage than CuSO_4_ which may offer benefits in vitamin stability when included in a vitamin-trace mineral premix (Luo et al. 2005).

Previous findings have observed increases in growth performance of nursery pigs with additions of 100 to 250 mg/kg Cu, regardless of source (Fry et al. 2012; Huang et al. 2015; Espinosa et al. 2017). Cromwell et al. (1989) observed that feeding 150 mg/kg Cu was 75% as effective at increasing growth as feeding 250 mg/kg. However, other literature indicates that 125 mg/kg was equally effective in stimulating growth as 250 mg/kg Cu (Stahly et al. 1980). More recent research has observed that feeding 160 mg/kg or 200 mg/kg of TBCC resulted in similar weight gain and G:F but greater performance than pigs fed less than 80 mg/kg Cu (Lin et al. 2020). Therefore, 150 mg/kg of Cu was added to diets for the respective feeding strategy within the current trial.

In nursery diets, it is common in some countries to feed high levels of Zn during the first two or three weeks after weaning as it has been shown to promote growth rate and increase stool firmness (Bonetti et al. 2021; Faccin et al. 2023; Tang et al. 2024). However, it is important to evaluate Cu effects when these levels of Zn are included within the diet because of possible Cu and Zn interactions. Some research indicates there are no additive effects when high levels of Zn and Cu are used together compared to using only high levels of Zn (Smith et al. 1997). However, Pérez et al. (2011) indicated that 3000 mg/kg Zn (from ZnO) and 100 mg/kg of Cu (from an amino acid complex) or 250 or 315 mg/kg diet as CuSO_4_ were additive in promoting the growth of weaned pigs. Other studies have indicated minimal additive improvement when feeding high levels of Zn and Cu together (Hill et al. 2000; Shelton et al. 2011). To further evaluate the use of high levels of Cu and Zn together, 2000 to 3000 mg/kg of Zn were included within the diet of the current trial. Hill et al. (2000) observed an improvement in G:F during the second week after weaning when high levels of Zn and Cu were fed together. In the current study, no differences in G:F were observed, but rather the improvement in ADG in phase 2 appears to be the result of the increase in ADFI. Similarly, Zhu et al. (2011) observed increased ADFI with high levels of added Cu.

While numerous studies have characterized high levels of added Cu used within the nursery phase, these levels of Cu can also be used to increase growth performance in finishing pigs (Braude and Hosking 1982). However, results are variable with some trials indicating Cu level did not influence overall performance (Carpenter et al. 2019; Bradley et al. 2023). Of the trials that have observed a response from an additional 75 to 150 mg/kg of Cu, many are similar to the current study with increased growth performance in the grower period (Hastad et al. 2001; Coble et al. 2017, 2018). However, others have observed improved gain in the finishing period as well (Davis et al. 2002; Zhao et al. 2014). Zheng et al. (2018) observed a tendency for CuSO_4_ to have a greater ADG than pigs fed TBCC, unlike the current trial, where Cu source did not affect ADG when fed at 150 mg/kg, which is similar to results of Hastad et al. (2001) and Coble et al. (2017). The gain response observed from Cu is often a function of feed intake (Li et al. 2008). Coble et al. (2017, 2018) observed an increase in ADFI when feeding additional Cu in the diet in the initial few weeks of the growing period. Other research has observed an improvement in G:F when high levels of Cu were fed (Barber et al. 1955; Davis et al. 2002; Hernández et al. 2008). In the current trial, the improvement in gain seems to primarily be associated with feed intake, however G:F was slightly improved for pigs fed the additional 150 mg/kg of Cu in the growing period.

Some previous research agrees with the observed improvement in final BW and carcass weight with 75 to 150 mg/kg of Cu due to improvements in growth (Coble 2017). In addition, some have observed increases in loin depth, lean percentage, and dressing percentage (Myres and Bowland 1973a; Coble et al. 2017). Myres and Bowland (1973b) thought copper may alter adipose tissue fatty acid composition by disrupting the balance between triglyceride lipolysis and free fatty acid esterification and therefore influencing carcass quality. However, these responses have not been consistent, as other studies have reported no effects on carcass characteristics (Bowland 1990; Coble et al. 2018).

In conclusion, these data suggest that in the nursery (Exp. 1), when 2000 to 3000 mg/kg of added Zn are used, ADG and ADFI can be improved by the addition of 150 mg/kg of Cu in the diet from d 10 to 24. The improvement in ADG and ADFI within phase 2 from the addition of 150 mg/kg of Cu did not influence overall performance, removals, or mortality. However, pigs fed 150 mg/kg Cu in phases 2 and 3 had numerically greater ADG compared with those fed other feeding strategies. In grow-finish diets (Exp. 2), these data suggest there were no differences in pig performance due to Cu source when fed at 150 mg/kg. However, feeding an added 150 mg/kg of Cu regardless of source improved growth performance, with the greatest response observed in the early growing period.

## List of Abbreviations

ADG: average daily gain
ADF: acid detergent fiber
ADFI: average daily feed intake
BF: backfat
BW: bodyweight
CP: crude protein
CuSO_4_: copper sulfate
DM: dry matter
G:F: Gain:feed ratio
HCW: hot carcass weight
TBCC: tribasic copper chloride
